# Bioelectrical impedance analysis-derived skeletal muscle mass index versus computed tomography for the detection of muscle mass reduction in patients with gastrointestinal cancer: a cross-sectional study

**DOI:** 10.3389/fonc.2026.1769615

**Published:** 2026-02-23

**Authors:** Bo Gao, Qinggang Yuan, Hao Zhang, Wenqing Chen, Xiangrui Li, Xiaotian Chen

**Affiliations:** 1 Department of Clinical Nutrition, Nanjing Drum Tower Hospital, Affiliated Hospital of Medical School, Nanjing University, Nanjing, China; 2 Department of Gastrointestinal Surgery, Xuzhou Central Hospital, Xuzhou, China; 3 Department of Clinical Nutrition, Jinshan Hospital of Fudan University, Shanghai, China

**Keywords:** bioelectrical impedance analysis, CT scan, gastric cancer, muscle mass, nutrition

## Abstract

**Background:**

The aims of this study were to assess the precision of bioelectrical impedance analysis (BIA) in evaluating muscle mass and to establish a population-specific cutoff value for identifying muscle mass reduction in a Chinese population with gastric cancer.

**Methods:**

A total of 163 patients with gastric cancer were enrolled. Skeletal muscle mass was measured at the L3 level using computed tomography (CT) scans. Muscle mass was concurrently evaluated using BIA. The correlations of muscle mass between CT and BIA methods were assessed. Data consistency was analyzed by the intraclass correlation coefficient (ICC). The optimal cutoff value of the BIA-derived skeletal muscle index (SMI) for identifying muscle mass reduction was determined by receiver operating characteristic (ROC) curve analysis.

**Results:**

The mean skeletal muscle mass measured by CT and BIA was 118.81 ± 24.54 cm^2^ and 25.11 ± 4.37 kg in overall patients, respectively. The mean SMI measured by BIA was 9.42 ± 1.09 kg/m^2^. SMI measured by CT was correlated with that measured by BIA (*r* = 0.727, *p* < 0.001). The ICC between the two methods was 0.903, indicating a satisfactory consistency. The optimal BIA-derived SMI cutoff values for identifying muscle mass reduction were 9.46 kg/m² for men and 8.72 kg/m² for women in this population.

**Conclusions:**

Muscle mass assessed by BIA showed a high correlation and satisfactory consistency with that measured by CT scan.

## 1 Introduction

Gastric cancer remains a serious health issue with high mortality rates (1). Sarcopenia, defined by the progressive loss of skeletal muscle mass and a decline in muscle strength, has been a major focus of research in recent years (2). Previous studies have demonstrated that sarcopenia serves as an independent risk factor in several malignancies (3). The comprehensive diagnosis of sarcopenia involves muscle mass and function reduction (3, 4). Muscle mass reduction measured by multiple techniques is an essential part in highlighting sarcopenia risk. Since the muscle mass reduction criteria varied across different ethnic groups and disease states, it is essential to identify hallmarks for muscle mass reduction in Chinese patients with gastric cancer to guide appropriate treatment strategies and provide optimal nutritional support. In addition, early identification prior to surgery and post-treatment follow-up to monitor muscle mass reduction are necessary to improve prognostic outcomes and ensure individualized supportive care.

Identification of muscle mass mainly relied on body composition assessment using various medical techniques, including computed tomography (CT), magnetic resonance imaging (MRI), dual-energy x-ray (DXA), and bioelectrical impedance analysis (BIA) (5–7). Among them, CT scan has been widely regarded as the gold standard for body composition assessment in most research (8). Skeletal muscle index (SMI), one of the essential diagnostic hallmarks of muscle mass reduction, is calculated from skeletal muscle mass at the L3 slice level of the CT scan and body height (9). However, the use of CT scan in evaluating skeletal muscle has several limitations, such as radiation exposure, high cost, and sophisticated operation and measurement procedures (10). Consequently, the application of CT for body composition assessment is largely confined to clinical settings, particularly during postoperative routine follow-up. Therefore, it is warranted to investigate alternative methods that are easy to perform and radiation-free and that provide comparatively accurate skeletal muscle measurement to complement or potentially reduce reliance on CT imaging.

The method of BIA is a non-invasive, radiation-free approach that estimates body composition by measuring the electrical impedance of current passing through different body tissues (11, 12). This technique uses an electrode system placed on the body surface to send a low-level measurement signal to the human body, detect the corresponding electrical impedance and its changes, and then obtain relevant physiological and pathological information. Its non-invasive, harmless, cost-effective, and user-friendly nature makes it suitable for routine body composition assessment in clinics (13). However, as BIA indirectly estimates human body composition data, its accuracy needs to be verified. In a cross-sectional study, Gao et al. demonstrated the accuracy of BIA in measuring visceral fat area compared with CT scans, supporting its use in screening for visceral obesity among patients with gastric cancer (14). Nevertheless, the accuracy of BIA in assessing muscle mass and its role in screening sarcopenia warrants further validation. Therefore, the aims of the present study were to investigate the accuracy of BIA for muscle mass evaluation and to establish a cutoff value for BIA-based muscle measurement in identifying muscle mass reduction in Chinese patients with gastric cancer.

## 2 Methods

### 2.1 Patients and methods

Patients with newly diagnosed primary gastric cancer between the ages of 18 and 80 years old, who were admitted to Nanjing Drum Tower Hospital between January 2017 and January 2019, were consecutively enrolled. All participants had no prior history of surgery, chemotherapy, or radiotherapy. The exclusion criteria included patients with severe edema, cardiac or renal failure, and cirrhosis; those who are using diuretics; pregnant patients; unconscious patients; those who were unable to cooperate or stand still; those who refused to participate; and those with unmeasurable skeletal muscle mass by CT. This observational cross-sectional study was in accordance with the Declaration of Helsinki, and written informed consent was obtained from enrolled participants at the time of hospital admission for the use of their clinical data and biological samples in future observational research. This study was approved by the Ethical Committee of Nanjing Drum Tower Hospital (2023-149-01) and covered the secondary use of previously collected clinical and imaging data for the present research purpose.

### 2.2 Baseline information

The baseline clinical characteristics of patients were collected, including age, sex, body weight, body height, and body mass index (BMI), which is calculated as body weight in kilograms (kg) divided by body height squared (m²). Additionally, tumor stage [classified according to American Joint Committee on Cancer (AJCC) criteria (15)], tumor tissue type, and comorbidities were also documented. Laboratory tests were conducted using fasting blood samples collected on the morning of admission.

### 2.3 Skeletal muscle mass measurement by CT scan

Patients enrolled underwent a CT scan within 24 h of admission. The CT scan method for skeletal muscle assessment has been described previously (16). In brief, a single slice at the L3 level was selected and analyzed using MATLAB (MathWorks, USA). Body tissue with Hounsfield unit (HU) ranging from −29 to 150 was considered skeletal muscle, and its area was measured (17). The density of skeletal muscle was expressed as the average HU value. The SMI by CT (SMI-CT) was defined as the skeletal muscle mass area at the L3 level normalized to the square of body height [SMI = skeletal muscle mass at L3 (cm^2^) square of body height (m^2^)] (17). The estimated fat-free mass (FFM) was estimated using the previously validated formula, which was 0.3 * (muscle L3 area) + 6.06 (18, 19). This procedure ensured accurate and standardized evaluation of skeletal muscle mass in the study participants.

### 2.4 Body composition assessment by BIA

The present research utilized multi-frequency eight-electrode bioelectrical impedance InBody 720 (InBody Co., Ltd., Seoul, Korea) to measure body composition. The BIA device underwent regular maintenance calibration according to the manufacturer’s recommendations and performed an automatic self-check calibration after each power-on. To minimize inter-operator variability, all BIA measurements were conducted by a single trained operator who was blinded to the CT-derived muscle measurements. Before the measurement, participants were asked to remove any metal objects from their bodies. Measurements were taken under fasting conditions with an empty bladder. Participants stood barefoot on the instrument, ensuring their feet are in complete contact with the foot-shaped electrodes on the panel. They hold the handles on both sides with both hands to ensure five fingers are in full contact with the test electrodes. Arms were required to be extended and maintained 15° apart from the body. The SMI by BIA (SMI-BIA) was defined as skeletal muscle mass (kg) measured by BIA/square of body height (m^2^).

### 2.5 Definition of muscle mass reduction in a Chinese population

Previous criteria for diagnosing muscle mass reduction were based on diverse ethnicities and health status, which may lead to overestimation or underestimation of muscle mass reduction in Chinese patients with gastric cancer. According to a large-scale study based on Chinese patients with gastric cancer in previous research, the standard of muscle mass reduction indicating sarcopenia risk adopted in the present study was SMI < 40.8 cm^2^/m^2^ in male patients and SMI < 34.9 cm^2^/m^2^ in female patients by CT scan at the L3 level (20).

### 2.6 Statistical analysis

Data were analyzed using SPSS (version 22.0, IBM, USA) and MedCalc (MedCalc Software, Ostend, Belgium). Continuous variables were presented as mean ± standard deviation (SD) if they were normally distributed, and their comparison was performed by an independent or paired *t*-test. For variables with skewed distributions, median (25th percentile, 75th percentile) was represented, and the Mann–Whitney *U* test was adopted for comparisons. Categorical variables were expressed as frequencies and percentages, and their comparisons were evaluated by χ^2^ test or Fisher’s exact test when appropriate. Correlations were assessed by Pearson’s or Spearman’s correlation coefficient. Reliability and agreement analysis between FFM based on CT and BIA were evaluated by the intraclass correlation coefficient (ICC) using a two-way mixed-effects model with absolute agreement. The consistency of the two measurements was evaluated using the Bland–Altman statistical method, with calculation of the 95% limits of agreement (95% LOA) (21). The performance of BIA-measured muscle mass in detecting muscle mass reduction was evaluated by the area under the receiver operating characteristic curve (AUROC). The optimal cutoff value estimation by BIA was obtained by the maximum Youden index (sensitivity + specificity − 1). Statistical significance was determined by a two-tailed *p-*value < 0.05. Based on the criteria proposed by Shrout and Fleiss, it was interpreted as poor reliability when ICC ranged from 0.00 to 0.49, satisfactory reliability from 0.50 to 0.74, and excellent reliability from 0.75 to 1.00 (21, 22).

## 3 Results

### 3.1 Baseline characteristics

**Figure 1:**
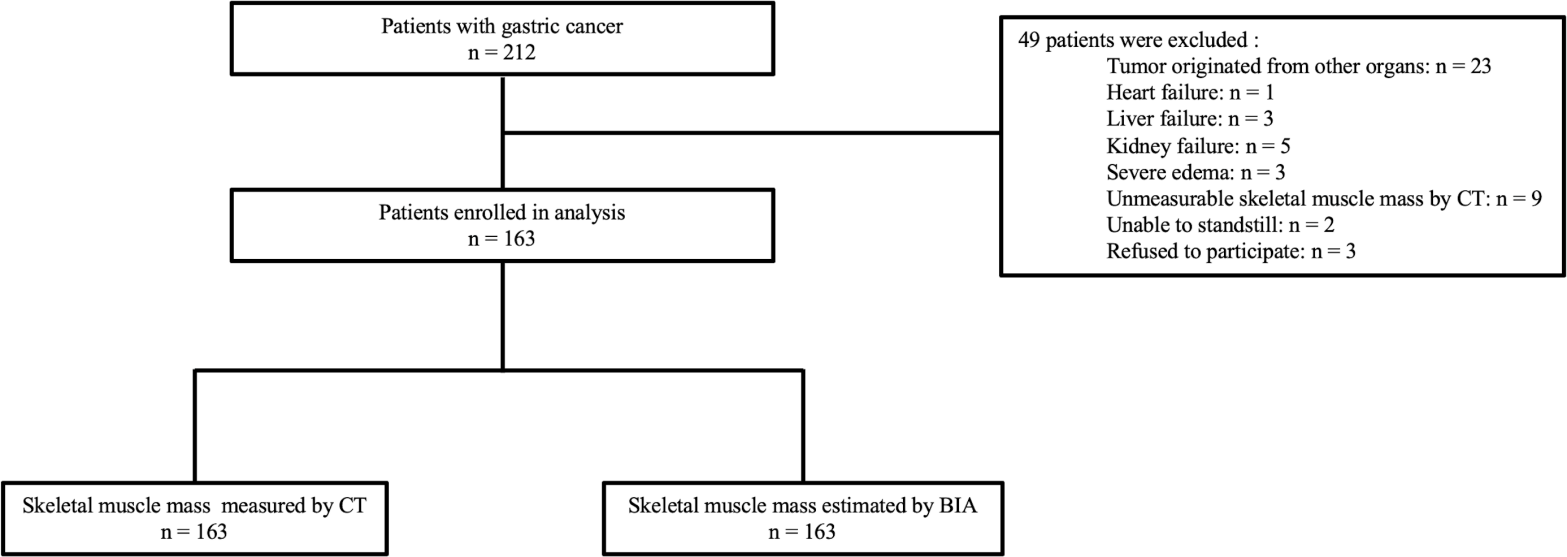
Flowchart of the research.

A total of 163 patients were finally enrolled and analyzed, including 44 female patients and 119 male patients. **Figure 1** shows the flowchart of the study. The average body weight was 62.14 ± 9.82 kg, and body height was 162.71 ± 7.33 cm, with a BMI of 23.42 ± 3.01 kg/m^2^. The majority of patients (125, with 76.7%) had adenocarcinoma tumor tissue type. The basic information and laboratory test results are listed in **Table 1**.

**Table 1:** Baseline characteristics.

Variable	Overall *N* = 163	Female *N* = 44	Male *N* = 119	*p*
Age (years)	60.97 ± 11.77	58.93 ± 12.71	61.72 ± 11.37	0.180
<65 years (%)	88 (54.0)	25 (56.8)	63 (52.9)	0.659
≥65 years (%)	75 (56.0)	19 (43.2)	56 (47.1)	
Gender (male, %)	119 (73.0%)	–	–	–
Body weight (kg)	62.14 ± 9.82	56.91 ± 8.33	64.07 ± 9.66	<0.001***
Body height (cm)	162.71 ± 7.33	155.26 ± 5.45	165.47 ± 5.88	<0.001***
BMI (kg/m^2^)	23.42 ± 3.01	23.61 ± 3.27	23.35 ± 2.92	0.623
<18.5	7 (4.3)	2 (4.5)	5 (4.2)	0.463
18.50–24.99	114 (69.9)	27 (61.4)	87 (73.1)	
25–29.99	38 (23.3)	13 (29.5)	25 (21)	
≥30	4 (2.5)	2 (4.5)	2 (1.7)	
Tumor stage (AJCC)				0.078
I	46 (28.2)	18 (40.9)	28 (23.5)	
II	34 (20.9)	8 (18.2)	26 (21.8)	
III	54 (33.1)	9 (20.5)	45 (37.8)	
IV	29 (17.8)	9 (20.5)	20 (16.8)	
Tissue type				0.766
Adenocarcinoma	125 (76.7)	34 (77.3)	91 (76.5)	
Signet ring cell carcinoma	8 (4.9)	3 (6.8)	5 (4.2)	
Others	12 (7.4)	2 (4.5)	10 (8.4)	
Unknown	18 (11.0)	5 (11.4)	13 (10.9)	
Diabetes (yes, %)	8 (4.9%)	4 (9.1)	4 (3.4)	0.274
Laboratory				
WBC (×10^9^/L)	5.60 ± 1.63	5.28 ± 1.53	5.71 ± 1.66	0.130
Hemoglobin (g/L)	123.71 ± 24.17	112.98 ± 18.84	127.67 ± 24.77	<0.001***
Platelet (×10^9^/L)	211.15 ± 74.68	200.89 ± 76.12	214.95 ± 74.11	0.287
Albumin (g/L)	38.42 ± 4.18	37.73 ± 5.03	38.67 ± 3.81	0.269
C-reactive protein (mg/L)	12.10 ± 26.45	18.28 ± 38.72	9.86 ± 20.05	0.180
CEA (ng/mL)	3.35 ± 7.99	1.56 ± 3.65	4.00 ± 8.99	0.015
CA125 (ng/mL)	15.34 ± 31.64	22.70 ± 55.82	12.60 ± 14.42	0.253
CA199 (ng/mL)	23.07 ± 50.84	25.59 ± 53.36	22.14 ± 50.09	0.709
CA724 (ng/mL)	8.77 ± 24.88	5.88 ± 14.70	9.85 ± 27.71	0.379
CA242 (ng/mL)	9.78 ± 17.54	11.63 ± 25.09	9.10 ± 13.81	0.426
Body composition by CT				
Skeletal muscle mass area (cm^2^)	118.81 ± 24.54	92.61 ± 13.20	128.50 ± 20.31	<0.001***
Skeletal muscle density (HU)	38.91 ± 7.38	35.59 ± 7.64	40.14 ± 6.92	<0.001***
Estimated FFM (kg)	41.70 ± 7.36	33.84 ± 3.96	44.61 ± 6.09	<0.001***
SMI-CT (cm^2^/m^2^)	44.63 ± 7.51	38.47 ± 5.47	46.90 ± 6.86	<0.001***
Muscle mass reduction (*n*, %)	66 (40.5)	28 (63.6)	38 (31.9)	<0.001***
Body composition by BIA				
FFM (kg)	43.29 ± 6.85	36.13 ± 3.93	45.94 ± 5.72	<0.001***
Skeletal muscle mass (kg)	25.11 ± 4.37	20.52 ± 2.38	26.81 ± 3.66	<0.001***
SMI-BIA (kg/m^2^)	9.42 ± 1.09	8.50 ± 0.77	9.76 ± 0.99	<0.001***

Remarks: * indicated for *p* < 0.05; ** indicated for *p* < 0.01; *** indicated for *p* < 0.001.

BMI, body mass index; WBC, white blood cells; CEA, carcinoembryonic antigen; CA125, carbohydrate antigen 125; CA 199, carbohydrate antigen 199; CA724, carbohydrate antigen 724; CA242, carbohydrate antigen 242; SMI, skeletal muscle index; SMI-CT, SMI measured by CT; SMI-BIA, SMI measured by BIA. Estimated FFM by CT was adopted from a formula by a previous research of FFM = 0.3 * (muscle L3 area) + 6.06.

### 3.2 Body composition measurement by CT scans and BIA

Each patient enrolled underwent a CT scan and a BIA 24 h after admission. Body composition analysis was performed as previously described. Through CT scan analysis, the average skeletal muscle mass area was 118.81 ± 24.54 cm^2^ with an average muscle density of 38.91 ± 7.38 HU. FFM estimated by CT according to previous studies was 41.70 ± 7.36 kg, 33.84 ± 3.96 kg in female patients, and 44.61 ± 6.09 kg in male patients. Normalized with body height, the SMI by CT scan, represented as SMI-CT, was 44.63 ± 7.51 cm^2^/m^2^. The number and incidence of muscle mass reduction in the present study were 66 and 40.5%, respectively. Through the BIA method, the measured FFM was 43.29 ± 6.85 kg and 25.11 ± 4.37 kg, respectively. The SMI calculated by the BIA method, represented as SMI-BIA, was 9.42 ± 1.09 kg/m^2^. The muscle parameters measured by CT or BIA between female and male patients were all significantly different (*p* < 0.001), indicating distinct muscle conditions between genders (**Table 1**).

### 3.3 Correlation of SMI-CT and SMI-BIA and consistency of FFM estimated by CT and that measured by BIA

The consistency between FFM, a marker highly indicative of the body’s muscle state, calculated by CT scan as above described was evaluated with that measured by BIA. The Pearson correlation coefficient for SMI between the two methods was 0.727 (*p* < 0.001), indicating a notable association (**Table 2**). The ICC for FFM across the two methods was 0.903, representing satisfactory consistency and reliability. With Bland–Altman analysis, the mean difference observed between the two techniques was 1.58 ± 4.23 kg, indicating that BIA overestimated muscle mass by 1.58 kg on average in overall patients (**Table 3**; **Supplementary Figure 1**). Over 95% of the data points were distributed within the LOA, indicating acceptable agreement at the statistical level. However, the 95% LOA were relatively wide (−6.71 to 9.88 kg), suggesting a non-negligible bias in individual-level measurements. This finding indicates that BIA cannot be considered interchangeable with CT for direct muscle mass quantification (**Supplementary Figure 1**).

**Table 2:** Correlation of SMI assessed by CT with that measured by BIA.

Variable	SMI-CT (cm^2^)	SMI-BIA (kg)	*r*	*p* for *r*
Overall *n* = 163	44.63 ± 7.51	9.42 ± 1.09	0.727	<0.001***
BMI (kg/m^2^)				
<25 *n* = 121	43.01 ± 6.68	9.15 ± 0.94	0.612	<0.001***
≥25 *n* = 42	49.27 ± 7.89	10.22 ± 1.12	0.814	<0.001***
Gender				
Female *n* = 44	38.47 ± 5.47	8.50 ± 0.77	0.581	<0.001***
Male *n* = 119	46.90 ± 6.86	9.76 ± 1.00	0.643	<0.001***
Age (years)				
<65 *n* = 88	46.28 ± 7.48	9.61 ± 1.14	0.723	<0.001***
≥65 *n* = 75	42.69 ± 7.10	9.20 ± 0.99	0.705	<0.001***
Tumor stage				
Early (I, II) *n* = 80	44.93 ± 8.05	9.32 ± 1.07	0.793	<0.001***
Late (III, IV) *n* = 83	44.33 ± 6.97	9.52 ± 1.11	0.677	<0.001***

Remarks: * indicated for *p* < 0.05; ** indicated for *p* < 0.01; *** indicated for *p* < 0.001. BMI, body mass index.

**Table 3:** Consistency of FFM assessed by CT with that measured by BIA.

Variable	CT-FFM (kg)	BIA-FFM (kg)	BIA-CT FFM (kg)	Paired *t*	*r*	ICC	95% LOA
Overall *n* = 163	41.70 ± 7.36	43.29 ± 6.85	1.58 ± 4.23	<0.001***	0.825	0.903	−6.71, 9.88
BMI (kg/m^2^)							
<25 *n* = 121	40.53 ± 6.57	42.27 ± 6.23	1.74 ± 4.35	<0.001***	0.770	0.869	−6.79, 10.28
≥25 *n* = 42	45.09 ± 8.47	46.23 ± 7.74	1.13 ± 3.88	0.045*	0.890	0.940	−6.46, 8.73
Gender							
Female *n* = 44	33.84 ± 3.96	36.13 ± 3.93	2.29 ± 3.56	<0.001***	0.692	0.744	−4.689, 9.27
Male *n* = 119	44.61 ± 6.09	45.94 ± 5.72	1.32 ± 4.44	0.001**	0.719	0.835	−7.38, 10.02
Age (years)							
<65 *n* = 88	43.54 ± 7.65	44.45 ± 7.21	0.91 ± 4.22	0.047*	0.840	0.912	−7.36, 9.18
≥65 *n* = 75	39.55 ± 6.41	41.93 ± 6.18	2.38 ± 4.13	<0.001***	0.785	0.879	−5.71, 10.47
Tumor stage							
Early (I, II) *n* = 80	41.77 ± 7.73	42.58 ± 6.55	0.81 ± 4.08	0.042*	0.849	0.911	−7.19, 8.81
Late (III, IV) *n* = 83	41.64 ± 7.04	43.97 ± 7.10	2.34 ± 4.26	<0.001***	0.818	0.900	−6.01, 10.69

Remarks: * indicated for *p* < 0.05; ** indicated for *p* < 0.01; *** indicated for *p* < 0.001. BMI, body mass index.

### 3.4 Subgroup analysis

Patients were stratified by gender, median BMI level, age, and tumor stage, and the analysis was performed within subgroups. For patients with different BMI levels, SMI was also correlated across groups, with Pearson’s *r* of 0.612 (low BMI) and 0.814 (high BMI; all *p* < 0.001). ICC of FFM (low BMI: ICC = 0.869; high BMI: ICC = 0.940) showed good consistency. For patients with different genders, SMI was also correlated between the two methods (female *r* = 0.581, male *r* = 0.643, all *p* < 0.001), and FFM consistency was satisfactory, with the following ICCs: female ICC = 0.74 and male ICC = 0.84. Similarly, a correlation between age and tumor stage was observed (**Table 3**).

### 3.5 Receiver operative characteristic analysis

**Figure 2:**
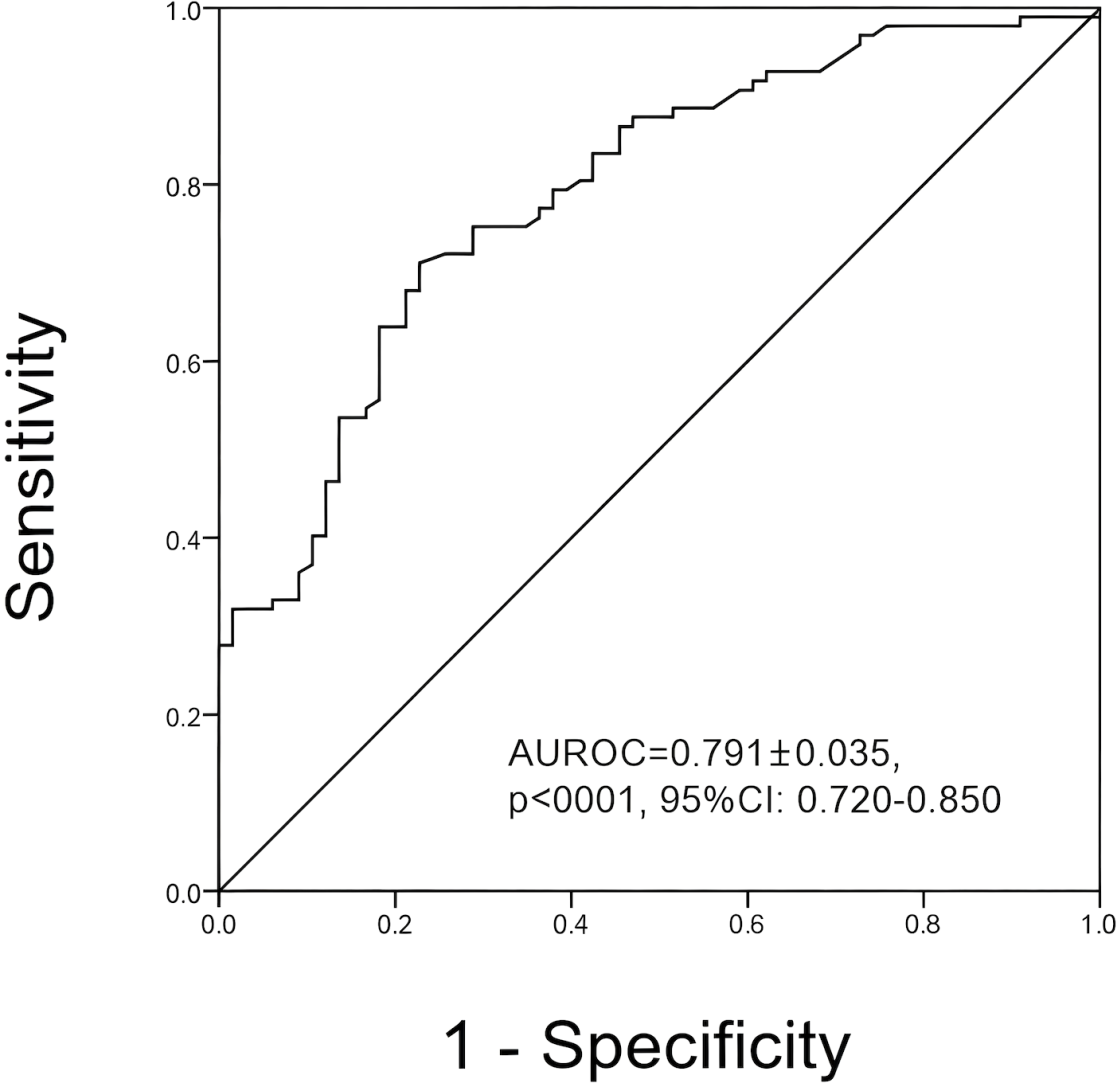
ROC plots of SMI-BIA in detecting muscle mass reduction in overall subjects. (The AUROC = 0.791 ± 0.035, *p* < 0.001, 95% CI: 0.720–0.850. The optimal cutoff value of SMI obtained by BIA in detecting muscle mass reduction was 9.39 kg/m^2^ in overall patients enrolled.).

In overall patients, SMI measured by BIA was capable of indicating muscle mass reduction as assessed by CT scan in the present study (AUROC 0.791 ± 0.035, *p* < 0.001, 95% CI: 0.720 to 0.850) (**Table 4**; **Figure 2**). The optimal cutoff value was 9.39 kg/m^2^ (sensitivity: 71.1%, specificity: 77.3%), based on the maximum Youden index, indicating that patients with SMI measured by BIA below 9.39 kg/m^2^ should be highly suspected of muscle mass reduction. SMI-BIA showed satisfactory performance for muscle mass reduction in subgroups stratified by BMI, gender, age, and tumor stage (all *p* < 0.05). Notably, the muscle condition was significantly different between genders. Sex-specific SMI-BIA cutoff values were obtained. The optimal cutoff values in the present study were 8.72 kg/m^2^ in female patients (AUROC: 0.792 ± 0.072, *p* < 0.001, 95% CI: 0.644 to 0.900, sensitivity 68.6%, specificity 76.5%) and 9.46 kg/m^2^ in male patients (AUROC: 0.749 ± 0.446, *p* < 0.001, 95% CI: 0.655, 0.819, sensitivity 76.5%, specificity 65.8%) (**Table 5**).

**Table 4:** AUROC of SMI by BIA in detecting muscle mass reduction.

Variable	SMI AUC ± SE (95% CI)	*p*
Overall	0.791 ± 0.035 (0.720, 0.850)	<0.001***
BMI < 25 kg/m^2^	0.726 ± 0.047 (0.637, 0.803)	<0.001***
BMI ≥ 25 kg/m^2^	0.931 ± 0.038 (0.818, 0.989)	<0.001***
Female	0.792 ± 0.072 (0.644, 0.900)	<0.001***
Male	0.749 ± 0.446 (0.655, 0.819)	<0.001***
Age < 65 years	0.830 ± 0.050 (0.736, 0.902)	<0.001***
Age ≥ 65 years	0.735 ± 0.060 (0.620, 0.830)	<0.001***
Early stage (I, II)	0.836 ± 0.047 (0.737, 0.910)	<0.001***
Late stage (III, IV)	0.759 ± 0.053 (0.653, 0.846)	0.015*

* indicated for *p* < 0.05; ** indicated for *p* < 0.01; *** indicated for *p* < 0.001.

**Table 5:** Sex-specific cutoff values of SMI by BIA for the detection of muscle mass reduction.

Variable	Cutoff value (kg/m^2^)	Sensitivity %	Specificity %
Overall	9.39	71.1	77.3
Female	8.72	68.6	82.1
Male	9.46	76.5	65.8

## 4 Discussion

This research discovered that the muscle mass estimated by the BIA method was associated with that measured by CT scan at the L3 slice level, both in the entire patient group and subgroup analysis cohorts in patients with gastric cancer among the Chinese population. The ICC was high (ICC = 0.903), indicating a reliability for the two methods in muscle mass evaluation. This conclusion was consistent with previous research conducted on patients with gastric cancer to assess the consistency of visceral fat area using these two methods (14). Moreover, the ROC results reflected the ability of BIA-derived measurements to approximate CT-defined muscle mass reduction using CT as the reference standard. Nutritional assessment and support are important issues in clinics and warrant much more attention. This study provided a simple method to routinely evaluate patients’ nutritional status, thereby enabling more targeted nursing guidance.

Sarcopenia is characterized by the depletion of skeletal muscle mass, strength, and function, and it is particularly prone to occur in both patients with tumor and elderly patients (2). Currently, there is consensus that sarcopenia could act as a potential negative risk factor in the prognosis of various malignancies (23). Gastric cancer is a widespread cancer type that has high global morbidity and mortality rates. Despite the overall prognosis of gastric cancer gradually improving due to the advancement of early diagnosis and treatment, the involvement of the digestive tract leads to poor absorption of nutrients, which makes patients particularly susceptible to a malnutrition state. Aoyama et al. proposed that malnutrition conditions, including sarcopenia, during multidisciplinary therapy was attributed to poor physical activity, quality of life, and toxicity in chemotherapy, and eventually to poor survival (24). Hisada et al. also demonstrated that sarcopenia apparently influenced the prognosis of gastric cancer, even in the early stage, receiving endoscopic submucosal dissection (25). A European study conducted by Paolo et al. concluded that nutritional support in the palliative care period for patients with cancer was effective in improving quality of life. The research by Mulazzani et al. concluded that the tolerance and response to chemotherapy or radiotherapy were better in well-nourished patients with tumor (26). Therefore, early identification of muscle mass reduction is conducive to the selection of appropriate treatments and reasonable nutritional support for patients with gastric cancer. Consequently, researchers were committed to the discovery of a detection method for early screening of muscle mass reduction that not only is appropriate for routine application but also ensures precise screening.

Although CT scans could serve as the gold standard for body composition assessment due to their accuracy, they still possess several drawbacks. Firstly, the radiation exposure to patients exerts potential health risks over repeated or long-term use during the follow-up period after anti-tumor therapy. Secondly, the comparatively higher expense, specialized software, and professional training requirements made them less accessible for routine measurement. In particular, these restrictions became even more pronounced in remote areas with limited equipment and expertise.

Previous studies have demonstrated that the BIA approach is effective but not entirely accurate in determining body composition. Boykin et al. compared off-season body composition changes between BIA and DXA in athletes and discovered that BIA was less sensitive in detecting segmental FFM (27). A Brazilian study conducted by Amaral et al. specifically identified reference percentiles for BIA parameters in healthy individuals (28). Moreover, Näsänen-Gilmore et al. established a predictive equation for BIA among Malawian young adolescents (29). Shchelykalina et al. concluded that the BIA equations were based on different populations (30). Additionally, the accuracy of BIA was influenced by the patient’s condition, such as disease entities, edema severity, gender, and ethnicity (30). Thus, to mitigate measurement inaccuracies associated with the limitations of BIA, it is essential to perform BIA measurements under standardized protocols and to establish population-specific reference standards for muscle mass reduction.

This study demonstrated correlations and reliability between muscle parameters measured by BIA and the gold standard of body composition assessment. This was consistent with the previous work carried out by Lai et al. (31). Moonen et al. validated the correlations between BIA with CT in fluid ratios and overhydration; however, the cutoff values and references remain unclear (32). The most widely used and authoritative criteria and cutoff values for diagnosing sarcopenia by the BIA method were derived from different populations and differed across ethnic groups (33). For instance, the European Working Group on Sarcopenia in Older People (EWGSOP) criteria for sarcopenia were SMI < 8.87 kg/m^2^ in male patients and < 6.42 kg/m^2^ in female patients by BIA (34). The Asian Working Group for Sarcopenia (AWGS) defined sarcopenia as SMI < 7.0 kg/m^2^ in male patients and < 5.7 kg/m^2^ in female patients (35). The study by Petermann et al. showed that the prevalence of sarcopenia varied significantly across different categorization and cutoff criteria (33). Moreover, the population cohort of the above standards was mainly based on normal young or older people. Direct adoption of such criteria may lead to overestimation or underestimation of sarcopenia risk in clinics for patients with gastric cancer. The criteria for muscle quantity decrease were specifically based on a Chinese cohort with gastric cancer from a large-scale study. The cutoff values in gastric cancers in a Chinese population were higher than the previous criteria based on the normal population, which indicated a higher probability of missed diagnosis in the clinics if the previous criteria were adopted directly to Chinese patients with gastric cancer, and patients with gastric cancer were more susceptible to muscle quantity reduction than normal people. This study provided a reference for sex-specific cutoff values of SMI by BIA, indicating muscle mass reduction specifically in patients with gastric cancer among the Chinese population. In future studies, prospective research incorporating both muscle mass assessment (via BIA and CT) and muscle function tests should be designed to conduct a more comprehensive analysis of sarcopenia and further validate the clinical value of these imaging and measurement methods.

In addition, the moderate detection performance observed in the ROC analysis may partly reflect the intrinsic differences in measurement principles and clinical roles of BIA compared with CT. It should be emphasized that BIA has the ability to approximate CT-defined muscle mass reduction rather than demonstrate diagnostic equivalence or interchangeability between the two methods. From a clinical perspective, the role of BIA should be interpreted within a stepwise and longitudinal assessment framework rather than as a direct replacement for CT-based muscle evaluation. In patients with newly diagnosed gastric cancer, CT already routinely performed for staging serves as an appropriate reference standard for confirming muscle depletion and risk stratification at baseline. BIA offers a practical, non-invasive, and repeatable method for routine postoperative screening and longitudinal monitoring of muscle status. Although BIA carries a risk of false-negative classification, particularly in patients with cancer-related inflammation or early cachexia, the clinical consequences of false-positive results are generally limited, whereas false-negative results can be mitigated by repeated assessments and integration with clinical judgment.

The use of BIA could enable the early identification of patients at risk of muscle mass reduction prior to gastric cancer surgery. This facilitates individualized nutritional interventions and improves preoperative conditioning and postoperative recovery. Furthermore, routine postoperative BIA monitoring may allow for the timely adjustment of nutritional support, helping enhance functional recovery and reduce postoperative complications. Muscle mass reduction is linked to increased treatment-related toxicity. BIA-based screening and longitudinal monitoring of muscle status may assist in identifying patients at higher risk of poor chemotherapy tolerance. This prompts closer nutritional surveillance, early supportive care, and personalized treatment planning during therapy, and the influence of chemotherapy on muscle mass alteration warrants further investigation in future longitudinal studies.

The current study had some limitations. The study was a single-center investigation with a relatively small sample size. The BIA cutoff values were derived and tested within the same cohort without internal or external validation, which introduces a risk of optimism bias and limits the clinical generalizability of the proposed thresholds. These cutoff values should be considered preliminary. Additionally, some subgroup analyses were constrained by small sample sizes, which may have reduced statistical power. Thus, these findings should be interpreted as exploratory, and caution is advised when extrapolating the results to broader populations. Further multicenter studies with larger samples should be carried out to validate the current conclusions. Next, given the different measurement principles underlying CT and BIA, as well as the use of a conversion formula to harmonize measurement units, some degree of measurement inconsistency between the two methods cannot be entirely excluded. In this study, ICC was used to assess the consistency between CT- and BIA-derived measurements, rather than to imply methodological equivalence or interchangeability. Importantly, agreement and potential systematic bias between the two methods were primarily evaluated using Bland–Altman analysis, which served as an approach for assessing measurement agreement. Despite these limitations, BIA offers practical advantages, including no radiation exposure, convenience, and low cost. Given its acceptable agreement with CT in this study, BIA may still have clinical value for routine muscle mass assessment, although further longitudinal validation is required.

## 5 Conclusion

In conclusion, this research discovered that BIA-derived SMI showed satisfactory predictive approximation against CT-based assessment in identifying muscle mass reduction. The cutoff value of SMI by BIA was obtained sex-specifically, indicating muscle mass reduction, thus providing a reference in the clinic for better healthcare. The BIA method is particularly suitable for routine screening and follow-up due to its advantages over CT scan in the body composition assessment discussed.

## Data Availability

The data was available at the corresponding authors upon reasonable request.

## Supplementary material

Supplementary Figure 1 Bland–Altman plot for comparing the two methods. (The mean bias between the two measurements was 1.58 ± 4.23 kg. 95% limits of agreements ranged from −6.71 to 9.88 kg.)
