# Supplementary figures and images for: Bioelectrical impedance analysis-derived skeletal muscle mass index versus computed tomography for the detection of muscle mass reduction in patients with gastrointestinal cancer: a cross-sectional study
Source: Front Oncol. 2026 Feb 23;16:1769615. doi: 10.3389/fonc.2026.1769615 (PMC12967928; doi:10.3389/fonc.2026.1769615)

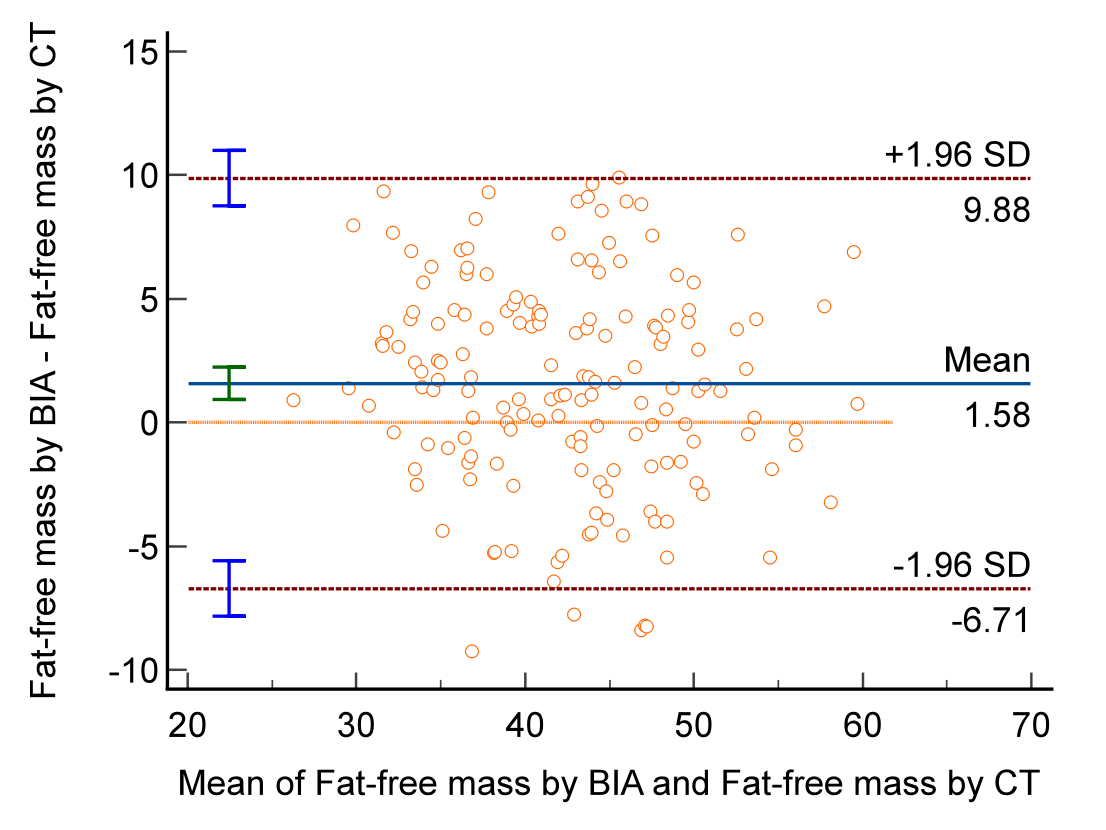

Supplement: Supplementary Figure 1 — Bland–Altman plot for comparing the two methods. (The mean bias between the two measurements was 1.58 ± 4.23 kg. 95% limits of agreements ranged from −6.71 to 9.88 kg.) [file Image1.tif]
